# Complete Genome Sequence of *Leptospira interrogans* Serovar Bratislava, Strain PigK151

**DOI:** 10.1128/genomeA.00678-15

**Published:** 2015-06-25

**Authors:** David P. Alt, Jennifer H. Wilson-Welder, Darrell O. Bayles, Caroline Cameron, Ben Adler, Dieter M. Bulach, Torsten Seemann, Michael J. Lehane, Lee R. Haines, Alistair C. Darby, Neil Hall, Alan D. Radford, Richard L. Zuerner

**Affiliations:** a National Animal Disease Center, Infectious Bacterial Diseases Research Unit, Ames, Iowa, USA; b Department of Biochemistry and Microbiology, University of Victoria, Victoria, British Columbia, Canada; c Departments of Microbiology and Biochemistry and Molecular Biology, Australian Research Council Centre of Excellence in Structural and Functional Microbial Genomics, Monash University, Clayton, Victoria, Australia; d Victorian Bioinformatics Consortium, Monash University, Clayton, Victoria, Australia; e Department of Vector Biology, Liverpool School of Tropical Medicine, Liverpool, United Kingdom; f Institute of Integrative Biology, University of Liverpool, Liverpool, United Kingdom; g Institute of Infection and Global Health, University of Liverpool, Liverpool, United Kingdom

## Abstract

*Leptospira interrogans* serovar Bratislava infection occurs in multiple domestic and wildlife species and is associated with poor reproductive performance in swine and horses. We present the complete genome assembly of strain PigK151 comprising two chromosomes, CI (4.457 Mbp) and CII (358 kbp).

## GENOME ANNOUNCEMENT

Worldwide, leptospirosis is one of the most widespread zoonoses. The genus *Leptospira* contains pathogens serologically classified into over 250 serovars, intermediate pathogens, and saprophytes with genetic classification into 21 different species (1–3).

*L. interrogans* serovar Bratislava has been isolated from both free-living and domestic species (4), swine with reproductive problems (5–7), dogs with renal disease (8), and horses (4, 9). In horses, poor reproductive performance has been linked with seroreactivity (10).

Current complete genome sequences have been published for three strains of *L. interrogans*, all from human clinical cases, representing serovars Lai and Copenhageni. Here, we report the genome sequence of *L. interrogans* serovar Bratislava strain PigK151 comprising two chromosomes, CI (4.457 Mbp) and CII (358 kbp), obtained from a sow’s kidney in Iowa in 1989.

High-quality genomic DNA was isolated from 5 × 10^10^ to 2 × 10^11^ pelleted *L. interrogans* following Marmur (11) with modifications. The pellet was suspended in 5 m of 50 mM Tris-EDTA and frozen at −70°C for 1 h. Room-temperature lysozyme solution was added with mixing; the cells were thawed and then placed on ice for 45 min. One milliliter of lysis buffer (950 µl 0.5% SDS, 50 mM Tris pH 7.5, 10 mM EDTA; and 50 µl of 20 mg/ml proteinase K, 50 mM Tris pH 8.0, 1 mM CaCl_2_) was added and mixed at 50°C for 1 h. Three rounds of extraction using Phase Lock Gel (5Prime) treatment with RNace-It (Agilent Technologies, Inc.) and a final chloroform extraction were performed.

Sequencing was performed using both Illumina HiSeq and Roche FLX-Titanium chemistry. Libraries were prepared according to manufacturers’ directions. A fully closed genome consisting of two chromosomes was assembled using MIRA version 3.4.0 (12) coupled with the Roche gsAssembler version 2.8. MIRA assemblies were hybrid assemblies comprising Roche FLX sequencing reads and Illumina paired-end reads providing 40× and 55× genome coverage, respectively. Roche gsAssembler assemblies used only Roche sequencing data obtained from GS FLX shotgun, GS FLXplus, and GS FLX Titanium large-insert (2.7 kb and 3.7 kb) mate pair sequencing reads. Illumina sequencing data consisted of 2 × 54-bp paired-end reads. Genome sequencing gaps were closed using either Gap Resolution version 1.2.2 (U.S. Department of Energy, Joint Genome Institute; http://jgi.doe.gov/data-and-tools) on the gsAssembler or by primer design followed by PCR amplification and Sanger sequencing. Editing was performed using GAP5 (13), from the Staden Package. The chromosomes were given a final polish by using SEQuel (14) to map Illumina reads back at approximately 180× total coverage to correct any remaining base calling and pyrosequencing errors in the assembly.

Annotation was performed using the NCBI Prokaryotic Genome Annotation Pipeline. The genome comprises two chromosomes: CI (4.457 Mbp) contains 3,486 coding sequences, 37 tRNAs covering all 20 amino acids, a single copy of the 5S rRNA, and two copies of both 16S and 23S rRNA; and CII (358 kbp) contains 287 coding sequences. Availability of additional complete genomic sequence data will enable better molecular comparisons and enhance the understanding of leptospiral pathogenicity.

### Nucleotide sequence accession numbers.

The annotated assembly is available in GenBank under the accession numbers CP011410 (CI) and CP011411 (CII).
